# Matrix metalloproteinase-12 in arterial diseases: context-dependent mechanisms of vascular remodeling and therapeutic implications

**DOI:** 10.3389/fcvm.2026.1825585

**Published:** 2026-06-18

**Authors:** Yifan Luo, Jiarong Wang, Jichun Zhao

**Affiliations:** 1 Division of Vascular Surgery, Department of General Surgery, West China Hospital, Sichuan University, Chengdu, Sichuan Province, China; 2 West China Lecheng Hospital, Sichuan University, Qionghai, China

**Keywords:** abdominal aortic aneurysm, aortic dissection, atherosclerosis, elastin-derived peptides, extracellular matrix, macrophage, MMP-12, vascular remodeling

## Abstract

Maladaptive vascular remodeling is a shared feature of diverse arterial disorders and arises from a self-reinforcing interplay between inflammation and extracellular matrix (ECM) turnover. Matrix metalloproteinase-12 (MMP-12), also known as macrophage elastase, has emerged as a context-dependent mediator within this crosstalk. This review synthesizes current knowledge on the structure, function, and regulation of MMP-12, detailing its multifaceted roles across arterial diseases. Divergent observations across experimental models and clinical settings can be better understood when MMP-12 activity is interpreted in relation to the inflammatory milieu, temporal disease stage, and substrate landscape. Within this framework, MMP-12 can act as a matrix-degrading protease, an amplifier of inflammatory cell recruitment, a regulator of protease cascades, or, in selected settings, a homeostatic modulator of inflammatory resolution. Finally, we discuss the opportunities and translational challenges of MMP-12-targeted strategies, emphasizing that therapeutic interpretation should be constrained by disease context, evidence level, and model heterogeneity.

## 1 Introduction

As a leading cause of death and disability worldwide, vascular diseases impose a substantial burden on healthcare systems amid global aging. Aortic aneurysms and dissections affect approximately 1%–3% of the general population, with prevalence rising to ∼10% among the elderly (1). Arterial disorders such as atherosclerosis (AS), abdominal aortic aneurysm (AAA), and aortic dissection (AD) differ in anatomy and clinical presentation, yet they share core pathological features, including chronic vascular inflammation, extracellular matrix (ECM) remodeling, elastin fragmentation, and progressive loss of arterial wall integrity (2). Within the protease network governing ECM remodeling, the matrix metalloproteinase family is repeatedly implicated. Among these enzymes, MMP-12 has attracted particular attention due to its potent elastolytic activity, distinct substrate preferences, and cellular source specificity. Beyond its classical role in matrix degradation, MMP-12 contributes to a broad array of pathological processes via its proteolytic activity. Emerging evidence has implicated MMP-12 as a coupling node that links ECM turnover to inflammatory amplification and protease cascade activation, thereby shaping maladaptive vascular remodeling (3–5).

However, the precise contribution of MMP-12 to vascular pathogenesis remains debated. While substantial evidence supports pathological roles of MMP-12 in arterial diseases, alternative studies indicate neutral or even protective effects under specific conditions (6, 7). This inconsistency reflects a broader limitation in the field. MMP-12 has often been interpreted either as a macrophage marker or as an elastase, rather than as an effector whose function depends on the inflammatory milieu, temporal disease stage, and substrate landscape, with additional consideration of lesion architecture, cellular source, and the specific model used to generate the evidence.

Accordingly, this review synthesizes current knowledge on the biology, regulation, and pathological roles of MMP-12 in arterial disease. We first summarize its structural and functional properties, then evaluate evidence for its involvement in AS, AAA, AD, and related vascular remodeling processes. We place particular emphasis on evidence hierarchy, model-dependent interpretation, and mechanisms extending beyond simple elastin degradation, with the aim of defining when MMP-12 should be viewed as a pathogenic effector, a disease-associated biomarker, or a context-dependent modulator of vascular remodeling. Finally, we discuss the translational barriers that must be addressed before MMP-12-targeted strategies can be clinically interpreted.

## 2 Biology of MMP-12

MMPs, or matrixins, belong to the metzincin superfamily of zinc-dependent endopeptidases and are capable of degrading a broad spectrum of ECM components (8). They are integral to both physiological and pathological processes, including embryonic development, morphogenesis, wound healing, inflammatory responses, and tumor metastasis. Based on substrate specificity, gene structure, and functional characteristics, MMPs are commonly categorized into six major subclasses: collagenases, gelatinases, stromelysins, matrilysins, membrane-type MMPs, and others. Despite their functional diversity, they share a conserved modular architecture comprising an N-terminal propeptide domain, a metalloproteinase catalytic domain, a variable-length hinge region, and a C-terminal hemopexin-like domain (9).

Within the MMPs, MMP-12 is distinguished by its potent elastinolytic activity. It was first identified in the conditioned medium of mouse peritoneal inflammatory macrophages. Like other MMPs, the *MMP12* gene is located on human chromosome 11q22.3 (10). Subsequent research by Shapiro et al. confirmed that both recombinant and native MMP-12 efficiently degrade insoluble elastin—the principal component of the elastic laminae in arterial media (11). Within the cardiovascular system, MMP-12 is derived mainly from macrophage-lineage cells. Human carotid plaque data indicate that MMP-12 is particularly enriched in macrophage subsets with tissue-remodeling features (12). Non-macrophage cardiovascular sources have also been reported, but in more restricted settings. VSMCs have been shown to produce functional MMP-12 during active vascular remodeling (13). When platelets are stimulated by collagen, they release active MMP-12, which then feeds back to promote further platelet activation (14). In the early phase after myocardial infarction, infiltrating neutrophils provide a transient MMP-12 source linked to neutrophil apoptosis and inflammatory resolution rather than sustained matrix degradation (15). The macrophage-dominated pattern extends beyond the cardiovascular system. In chronic inflammatory and remodeling diseases, MMP-12 is frequently enriched in disease-conditioned macrophages, including synovial CD163⁺ macrophages in rheumatoid arthritis, monocyte-derived cells in periodontitis, M2-like macrophages in chronic rhinosinusitis, and tumor-infiltrating macrophages (16–19). At the same time, epithelial cells, airway smooth muscle cells, fibroblasts, and some stromal cells can switch on MMP-12 expression under selected inflammatory or remodeling stimuli (20–22). Overall, macrophage-lineage cells remain the most consistent source, whereas other sources are inducible, disease-specific.

MMP-12 is typically secreted as an inactive 54-kDa zymogen that undergoes autolytic processing for self-activation, generating active forms of 45 kDa and 22 kDa (11). Structurally, full-length MMP-12 shares the modular organization typical of MMPs. Uniquely, however, shortly after activation, it undergoes autocatalytic shedding of the hemopexin-like domain while retaining robust elastinolytic capacity (23).

Functionally, MMP-12 exhibits significantly higher cleavage efficiency toward elastin than other MMPs. Compared to other elastin-degrading MMPs, MMP-12 employs a distinct catalytic mechanism. Unlike MMP-2 and MMP-9, which depend on fibronectin-like modules for elastin binding and digestion, MMP-12 interacts with elastin through distal exosites within its catalytic domain. This distinctive mechanism substantially enhances catalytic efficiency and substrate specificity, facilitating effective elastinolysis even in the absence of plasminogen activation (24–26).

Beyond elastin, MMP-12 exhibits broad substrate specificity toward diverse ECM components, including collagens, fibronectin, laminin, vitronectin, heparan sulfate proteoglycans, and chondroitin sulfate (27). This degradation capability enables MMP-12 to breach basement membranes, thereby facilitating macrophage infiltration into damaged tissues during inflammation. Other substrates of MMP-12 include myelin basic protein, pro-tumor necrosis factor-α, α-1 antitrypsin, chemokines, and plasminogen (28–31). Consequently, MMP-12 not only performs matrix-degrading functions but also directly regulates cellular behavior by processing diverse bioactive molecules.

## 3 MMP-12 in arterial remodeling and interpretive limits

Though substantial evidence links MMP-12 to arterial remodeling, the exact mechanisms of its action remain incompletely defined. In mice, *Mmp12* deficiency has been reported to disrupt aortic mechanical homeostasis even at a young age, leading to classic features of aging such as reduced elastic energy storage and decreased distensibility (32). Paradoxically, MMP-12 has also been reported as one of the most consistently upregulated senescence-associated secretory phenotype factors across multiple tissues with aging (33). Apparent discrepancies among published findings in arterial disease also suggest that the role of MMP-12 may be context-dependent and spatiotemporally specific. A potential explanation is that elevated MMP-12 may represent an adaptive response to injury. Within this framework, the timing, location, and magnitude of MMP-12 expression would determine whether its net impact favors inflammatory injury or tissue repair.

### 3.1 MMP-12 in atherosclerotic plaque remodeling

AS is a chronic arterial disease driven by dyslipidemia and chronic inflammation, characterized by the accumulation of fibrofatty plaques within the arterial wall. In murine hyperlipidemic models, high-fat feeding (HFD) increases aortic Mmp12 expression together with medial elastic lamina degradation, and LDL receptor-deficient mice show marked Mmp12 upregulation during advanced lesion formation (34, 35).

Stronger causal evidence comes from rabbit and genetic mouse models. In hypercholesterolemic rabbits, MMP-12 is absent from normal aorta but highly expressed in atherosclerotic plaques, predominantly in macrophage-derived foam cells, with a minor contribution from intimal VSMCs (36). A macrophage-specific transgenic rabbit model overexpressing human MMP-12 further demonstrates that MMP-12 can accelerate fatty-streak formation, microelastolysis, macrophage/VSMC accumulation, and later progression to larger aortic and coronary plaques (37–39). In ApoE-deficient mice, Mmp12 deletion reduces brachiocephalic plaque burden, macrophage accumulation, and other instability indices while increasing VSMC content (3). Similarly, long-term high-fat feeding studies indicate that Mmp12 deficiency reduces coronary atherosclerotic burden and sudden death (40). Mechanistically, MMP-12 appears to operate at the intersection of two processes rather than through elastin cleavage alone. First, it degrades elastin and basement membrane components, facilitating leukocyte entry and generating elastin-derived signals that can reinforce macrophage recruitment (36, 38, 39, 41). Second, its expression is concentrated in selected foam-cell macrophage subsets located near lipid core–fibrous cap interfaces and deep plaque regions, suggesting that MMP-12 is a marker of a specific inflammatory–proteolytic niche rather than a generic macrophage product (42–44).

Human evidence is broadly concordant but remains mainly associative. Plasma MMP-12 is associated with carotid intima-media thickness, ankle-brachial index, carotid plaque area, arterial stiffness, and cardiovascular disease in type 2 diabetes (4). In carotid endarterectomy specimens, MMP-12 is increased in smoker plaques with lower elastin content (45). Compared with those with stable plaques or no plaque, serum MMP-12 levels are elevated in patients with vulnerable plaques and are positively associated with subsequent cardiovascular and cerebrovascular events (46).

These findings support the view that MMP-12 marks macrophage-rich, elastin-remodeling, clinically active plaques. However, they do not establish whether circulating or tissue MMP-12 is a causal driver of plaque progression, a surrogate of inflammatory plaque burden, or a compensatory response to matrix injury.

This distinction is important because not all available data support a uniformly destabilizing role for MMP-12 in AS. Low elastin content in carotid plaques predicts ipsilateral stroke, yet plaque MMP-12 may correlate positively with elastin content in some cohorts (7). In addition, a biomechanical study suggests that elastin fragmentation may, under certain structural conditions, increase plaque–wall adhesion through fiber bridging rather than simply weaken plaque attachment (47). This finding argues against a strictly destabilizing role of MMP-12 and affords an initial glimpse into the complexity of its role. Such a dual effect is also consistent with recent views about plaque rupture: atherosclerotic lesions undergo a cycle of remodeling, including both stabilizing and destabilizing events, rather than becoming progressively weaker in a sequential manner (48).

Thus, in AS, MMP-12 should be framed as a biomarker of macrophage-rich matrix remodeling, rather than as a universally deleterious elastase. Current evidence therefore supports the mechanistic plausibility and preclinical targetability of MMP-12, but its clinical utility, causal contribution in human AS, and therapeutic tractability remain to be established.

### 3.2 MMP-12 in abdominal aortic aneurysm formation and progression

AAA is a degenerative vascular disease characterized by localized, irreversible, progressive dilatation that occurs most commonly in individuals over 65 years of age (49). In human AAA specimens, MMP-12 is expressed predominantly by infiltrating macrophages and is immunolocalized to residual medial elastin fragments, particularly within the transition zones adjacent to non-dilated normal aorta, suggesting a unique role for MMP-12 in aneurysm development (50). Consistent with this spatial pattern, pro-MMP-12 protein is markedly increased in AAA tissue relative to healthy aorta and is concentrated in macrophage-rich areas (51), while MMP-12 mRNA is progressively elevated from stable to ruptured AAA specimens (52). These findings link MMP-12 to macrophage-rich elastin-remodeling regions of aneurysmal tissue, but they remain largely associative.

Multiple studies utilizing diverse experimental animal models have yielded stronger causal evidence, but its interpretation is highly model-dependent. In the CaCl₂ adventitial injury model, Mmp12 deficiency reduces aneurysm formation, elastic fiber fragmentation, and macrophage infiltration, supporting a pathogenic role for MMP-12 in chronic inflammatory elastolysis (53). Similarly, macrophage-specific overexpression of human MMP-12 in transgenic rabbits amplifies adventitial inflammation, aortic dilation, and downstream protease activation involving MMP-2 and MMP-3, indicating that macrophage-derived MMP-12 can function as an upstream amplifier of elastin and collagen remodeling (54). Pharmacological inhibition further supports this interpretation in an atherosclerosis-associated Ang II model: selective MMP-12 inhibition with RXP470.1 reduces AAA incidence and rupture risk, limits elastin fragmentation, decreases macrophage infiltration, and promotes collagen deposition (5). Collectively, these models suggest that, in chronic macrophage-rich remodeling contexts with sustained elastin substrate availability, MMP-12 primarily behaves as a matrix-destructive and inflammation-amplifying protease.

This conclusion is not universal. In the porcine pancreatic elastase-induced model, Mmp12 deletion alone does not significantly suppress AAA formation, likely because exogenous elastase induces an acute proteolytic injury that rapidly disrupts elastic recoil and may obscure the contribution of downstream endogenous proteases (55). By contrast, the CaCl₂ adventitial application model used by Longo et al. is driven by sustained adventitial irritation, leading to chronic inflammation, progressive elastin fragmentation, and time-dependent dilation (56). More importantly, systemic Mmp12 deletion has recently been reported to worsen vascular inflammation and rupture susceptibility in a high-dose Ang II setting, accompanied by complement activation, neutrophil accumulation, and NET formation (6). This result does not simply contradict the pathogenic model of MMP-12; rather, it suggests that the net effect of MMP-12 changes when the dominant inflammatory program shifts from chronic macrophage-rich remodeling to an acute neutrophil/NET/complement-driven injury response. This interpretation is biologically plausible, although not yet directly proven in human AAA. In an arthritis model, Bellac et al. also demonstrated that MMP-12 exerts anti-inflammatory effects by inactivating C3a and C5a chemoattractants and clearing fibrin/actin within NETs, providing cross-disease mechanistic support (57). Similarly, early pharmacological inhibition of MMP-12 after myocardial injury has been associated with impaired neutrophil apoptosis and delayed inflammatory resolution (15).

Taken together, current literature supports a context-dependent rather than uniformly pathogenic model of MMP-12 in AAA. The apparently divergent AAA findings are best explained by differences in model design, inflammatory dominance, disease tempo, and intervention strategy. In chronic macrophage-rich models with sustained elastin substrate availability, MMP-12 appears predominantly deleterious by promoting elastolysis, macrophage recruitment, and downstream protease activation (5, 53, 54). By contrast, in acute rupture-prone settings characterized by neutrophil accumulation, NET formation, and complement activation, systemic loss of MMP-12 may expose homeostatic functions that are not captured by a simple matrix-degradation model (6).

Therefore, MMP-12 should be framed as a candidate mediator and biomarker of aneurysmal matrix remodeling, not as a generalizable therapeutic target for all AAA. Future studies should prioritize activity-based measurements, temporal sampling, lesion-region mapping, and inflammatory phenotype stratification to define which human AAA phenotypes are MMP-12-dependent, when MMP-12 inhibition would be beneficial, or whether systemic inhibition could be harmful in acute inflammatory or rupture-prone states.

### 3.3 MMP-12 in aortic dissection and medial degeneration

Recent studies have begun to implicate MMP-12 in AD and suggest its potential translational relevance. In a single-center study integrating tissue and serum analyses, Song et al. reported higher MMP-12 protein levels and enzymatic activity in the ascending aortic wall and serum of AD patients than in coronary artery disease patients and healthy controls. Immunohistochemistry localized MMP-12-positive cells to the deep medial layers at the dissection entry site, coinciding with elastic lamellar fragmentation and inflammatory cell accumulation. These observations provide direct evidence of *in situ* and circulating MMP-12 activity in AD (58). Similarly, Del Porto et al. localized MMP-12 expression to macrophage-rich inflammatory infiltrates at the edge of Stanford type A acute AD. In the same lesion regions, activated macrophages were accompanied by vascular endothelial growth factor and pro-inflammatory mediators, including IL-6, IL-8, and MCP-1, together with medial neovascularization and adventitial vasa vasorum remodeling (59). Collectively, these findings suggest that, in AD, MMP-12 may participate in a broader inflammatory–proteolytic–angiogenic niche rather than act as an isolated elastase. Within this context, MMP-12-associated elastin degradation may contribute to medial matrix weakening, whereas macrophage-derived cytokines and abnormal neovascularization may further amplify leukocyte recruitment and tissue fragility. This interpretation is consistent with the context-dependent framework of this review, in which the biological output of MMP-12 is shaped by the local inflammatory milieu, temporal stage, and structural substrate landscape. However, because these studies were cross-sectional and sampled established dissection tissue, they cannot determine whether MMP-12 activation precedes dissection, contributes to post-dissection remodeling, or reflects secondary inflammatory proteolysis after medial rupture.

Circulating MMP-12 has also been explored as a candidate biomarker for AD. Proietta et al. reported that serum MMP-12 levels in AD patients were significantly higher than in both healthy individuals and patients with carotid artery stenosis, and MMP-12 showed better discriminatory performance than general inflammatory markers such as IL-6 and IL-8 (60). A previous meta-analysis of circulating MMPs in acute AD also suggested that MMP-12 may be elevated in acute AD, although the evidence base was substantially smaller than that for MMP-9 (61). More recently, a large UK Biobank plasma proteomics study identified MMP12 as part of a four-protein panel associated with future AA/AD risk, with elevated MMP12 detectable years before diagnosis (62).

Taken together, current evidence supports a restrained interpretation that MMP-12 is best viewed as a candidate biomarker for AD and a plausible mediator of macrophage-rich medial proteolysis. The strongest support comes from spatial co-localization of MMP-12 with inflamed, elastin-fragmented regions of the dissected aorta and from elevated circulating MMP-12 in acute AD. By contrast, causal evidence remains weaker than in AAA and AS, because there are few AD-specific MMP-12-deficient or pharmacological intervention studies, and available human data are mostly small, cross-sectional, and heterogeneous in sampling time, comparator selection, and assay platform. Future work should prioritize clarifying the temporal kinetics of MMP-12 after dissection onset, establishing clinically actionable cutoffs, and testing incremental prognostic/diagnostic value over existing biomarkers and imaging pathways.

Key studies that have shaped the current understanding of MMP-12 in arterial disease are summarized in Table 1. By aligning principal findings with the limits of each line of evidence, the table provides a concise map of how MMP-12 has been linked to matrix destruction, inflammatory amplification, lesion remodeling, and, in selected settings, preservation of vascular integrity.

**Table 1:** Representative experimental and clinical evidence on MMP-12 in arterial diseases.

Disease	Study setting	Intervention/Analytical focus	Principal finding	Key limitation	Ref.
AS	HFD-fed ApoE^−/−^ Mouse	Mmp12^−/−^	Mmp12 deletion reduced plaque area, buried fibrous layers, macrophages, and increased SMC content	Murine model lifelong knockout; surrogate instability endpoints	(3)
AS	HFD-fed ApoE^⁻/⁻^ Mouse	Selective MMP-12 inhibition (RXP470.1)	RXP470.1 reduced plaque growth, macrophage invasion/apoptosis, and shifted plaques toward a more fibrous, less macrophage-rich phenotype	Short-term murine study; peptide PK, *in vivo* selectivity, systemic immune effects, and human efficacy remain unresolved	(120)
AS	Rabbits	Macrophage-specific hMMP-12 Overexpression	hMMP-12 overexpression accelerated lesion progression, macrophage/SMC accumulation, and elastic lamina destruction.	Transgenic rabbit evidence; supports pro-remodeling capacity but not direct human causality	(36, 38, 39)
AS	T2DM patients cohort	Plasma MMP-12 measurement	Higher plasma MMP-12 tracked with AS burden, arterial stiffness, and coronary events in T2DM patients	Special high-risk cohorts; association may reflect systemic inflammation or disease burden	(4)
AS	Carotid endarterectomy specimens with postoperative outcome follow-up	Plaque MMP-12⁺/CD68⁺ foam-cell macrophage ratio	MMP-12⁺ macrophage subset predicted vulnerable plaque features and adverse outcomes	Selected post-surgical cohort; prognostic association only, not causal evidence	(44)
AS	Human carotid plaque integrative study	Endogenous MMP-12 expression and plaque localization	MMP12 was enriched in symptomatic carotid plaques, localized to macrophage-rich elastin-remodeling regions, and promoted macrophage invasion.	MMP locus pleiotropy and advanced-lesion sampling limit causal attribution specifically to MMP-12.	(41)
AS	Ex vivo plaque delamination study in ApoE^−/−^ Mouse	Mmp12^−/−^	Mmp12 deficiency reduced internal elastic lamina fragmentation but also lowered plaque–wall adhesive strength	Ex vivo delamination surrogate; suggests context-specific interface effects	(47)
AS/AAA	HFD-fed ApoE^−/−^ Mouse	Mmp12^−/−^	Mmp12 loss did not reduce plaque growth, but markedly protected against medial elastin destruction, ectasia, and aortic dilation.	Lifelong knockout in murine model	(132)
AAA	Surgical human AAA specimens	MMP-12 mRNA/protein localization in aneurysm tissue	MMP-12 was produced by aneurysm-infiltrating macrophages and localized to residual medial elastin fragments, especially in active media and transition zones.	Spatially links MMP-12 to elastin degradation but does not prove necessity or sufficiency for AAA formation.	(50)
AAA	CaCl2-induced AAA Mouse	Mmp12^−/−^	Mmp12 deficiency attenuated aortic dilation, elastic lamellae disruption, and macrophage accumulation	Single CaCl₂ injury model	(53)
AAA	Elastase-perfusion AAA Mouse	Mmp12^−/−^	Aneurysm development was unaffected in mice with isolated deficiency of MMP-12	Acute exogenous elastase injury may bypass endogenous MMP-12 contribution	(55)
AAA	Rabbits with Carrageenan-induced adventitial inflammation	Macrophage-specific hMMP-12 Overexpression	hMMP-12 overexpression enhanced adventitial macrophage accumulation, medial/adventitial elastin loss, focal dilation, and MMP-2/-3 upregulation	Adventitial inflammation/overexpression model; implicates MMP-12 in elastolytic remodeling but not as a stand-alone driver of typical AAA	(54)
AAA	HFD-fed Apoe^−/−^ mice with Ang II infusion	Selective MMP-12 inhibition (RXP470.1)	RXP470.1 reduced AAA formation/progression and rupture-related death, preserved medial elastin, and promoted collagen-rich remodeling	Proof-of-concept in an single model	(5)
AAA	Ang II-infused ApoE^⁻/⁻^ mice	Mmp12^−/−^	Mmp12 deficiency increased aortic dilation, rupture/death, complement activation, NETosis, elastin damage, and reduced wall strength; complement blockade partially rescued the phenotype	Warns against indiscriminate MMP-12 inhibition but does not prove MMP-12 is protective in human atherosclerotic AAA	(6)
TAA/AAA	Human genetic causal-inference evidence	Circulating MMP-12 level	Genetically higher serum MMP-12 was associated with lower risks of both TAA and AAA	Circulating MR signal only; does not capture local aneurysm-wall MMP-12 activity or mechanism	(133)
AA/AD	Human population-level proteomic/genetic evidence	Circulating MMP-12 level	Circulating MMP-12 repeatedly emerged as a strong predictor of future AA/AD and was genetically linked to higher AA/AD risk in MR analyses.	Circulating protein/MR signal; does not define local active aneurysm-wall MMP-12 or validate intervention target	(62, 134, 135)
AD	Acute AD surgical Specimens	MMP-12 immunoreactivity/activity and tissue localization	MMP-12 immunoreactivity was detected mainly in cells located in the deeper region of the dissection entry site, where elastic fibers were disrupted and inflammatory cells were present	Small cross-sectional study with limited controls; indicates MMP-12 activation in acute AD but does not establish causality, cellular source, or diagnostic utility	(58)
AD	AD patients vs. risk-matched controls, with lesion histopathological assessment	Serum MMP-12 measurement and lesion inflammatory features	Serum MMP-12 was higher in AD than in risk-matched controls; AD lesions showed macrophage-rich inflammation and neovessel growth	Circulating biomarker and histological association; local MMP-12 source/activity and causal role in the aortic wall remain unproven	(59, 60)
TAAD	BAPN-induced murine TAAD model	IL-3 deficiency	IL-3 deficiency reduced TAAD, elastin degradation, macrophage inflammation, and MMP12 expression/activity; IL-3 induced macrophage MMP12 via IL-3Rβ–JNK/ERK–AP-1 signaling.	Supports an IL-3–driven inflammatory MMP12 axis, but does not establish MMP-12 as the sole causal effector in TAAD	(136)

AS, atherosclerosis; AAA, abdominal aortic aneurysm; AD, aortic dissection; TAA, thoracic aortic aneurysm; TAAD, thoracic aortic aneurysm/dissection; HFD, high-fat diet; Ang II, angiotensin II; MR, Mendelian randomization; NETs, neutrophil extracellular traps; SMC, smooth muscle cell.

## 4 Molecular regulation of MMP-12

**Figure 1:**
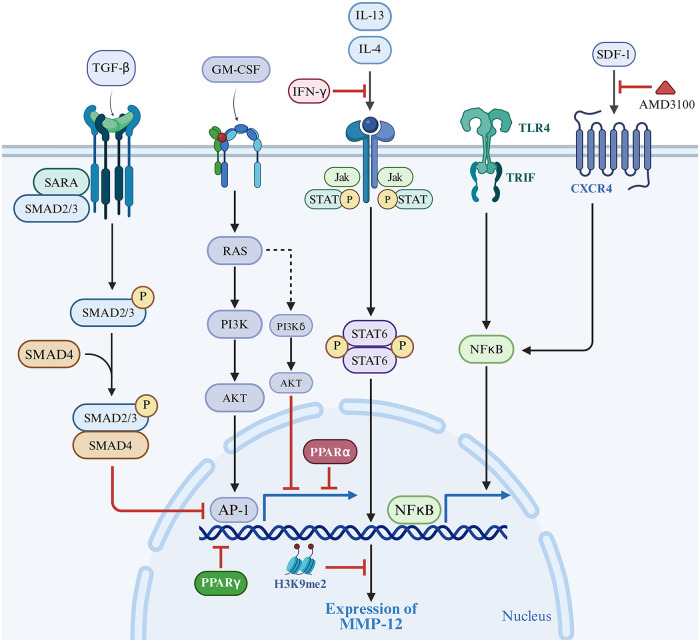
Upstream signaling pathways that regulate MMP12 transcription in vascular remodeling. Th2 cytokines (IL-4/IL-13) activate JAK–STAT6 signaling to induce MMP12 expression, whereas IFN-*γ* counter-regulates this program. GM-CSF enhances AP-1 activity through Ras–PI3K–Akt signaling. Innate immune and chemokine cues, including TLR4–TRIF and CXCL12 (SDF-1)–CXCR4, promote NF-*κ*B signaling that cooperates with AP-1 to drive MMP12 transcription. TGF-*β* signaling and nuclear receptors PPAR*α*/*γ* restrain MMP12 expression, while H3K9me2-mediated chromatin repression limits transcription factor access to the MMP12 promoter. Created with BioRender.com.

Expression of MMP-12 is controlled by a variety of signaling molecules, including Th1 and Th2 cytokines, growth factors, and metabolic regulators, which trigger specific intracellular signaling pathways. These pathways subsequently activate transcription factors that bind to the promoter region of the *MMP12* gene, thereby initiating its expression in response to environmental stimuli (Figure 1).

### 4.1 Th1/Th2 cytokines

The immune microenvironment is central to vascular homeostasis, with a wide range of cytokines and cell-interaction signals exerting a critical influence on MMP-12-centered vascular remodeling. In the context of AS-related inflammation, IL-1 (63), TNF-α (63), GM-CSF (64), M-CSF (64), IFN-*γ* (65), IL-4 (65), and transforming growth factor- β(TGF-β) (66) have all been implicated in the transcriptional or post-transcriptional regulation of MMP-12.

Among these signals, the opposite effects of IFN-*γ* and IL-4 have attracted particular attention. In an allogeneic aortic graft model, IL-4 increased MMP-12 expression in macrophages, whereas IFN-*γ* suppressed IL-4-induced MMP-12 expression (65).

Similar regulatory patterns have also been observed in broader macrophage-polarization studies, in which alternatively activated or M2-like macrophages can display higher MMP-12 expression than classically activated macrophages under selected experimental conditions (12, 63). More recent human macrophage data further show that IL-4 and IL-13, alone or together with tissue-derived mediators such as histamine, can enhance MMP12 expression in M2 macrophages (67). These findings place MMP-12 within a cytokine-sensitive macrophage remodeling program rather than within a purely constitutive protease response.

Evidence from human vascular lesions provides one possible link between this regulatory program and the divergent remodeling phenotypes of AS and AAA. Stenotic atherosclerotic plaques have been associated predominantly with Th1-type mediators, whereas human AAA samples have been reported to contain higher levels of Th2-associated cytokines, including IL-4, IL-5, and IL-10, together with lower levels of several Th1-associated mediators (68). This contrast was extended experimentally in an allogeneic aortic graft model, in which IFN-*γ* receptor deficiency shifted the inflammatory milieu toward IL-4 predominance. In that setting, grafts developed severe aneurysmal dilation, accompanied by increased MMP-9 and MMP-12 expression and enhanced collagenolytic and elastolytic activity; blockade or genetic absence of IL-4 prevented aneurysm formation and reduced proteolytic activity (65). These observations provide a mechanistic route by which a Th2-enriched environment may promote elastolytic macrophage activity and thereby favor aneurysmal remodeling.

These observations support the possibility that Th1/Th2 balance contributes to divergent vascular remodeling phenotypes by reshaping macrophage activation states and local protease expression. The Notch signaling pathway, a critical regulator of inflammatory responses, has been found to directly participate in maintaining Th2 bias. In the Ang II-infused *ApoE^⁻/⁻^* mice, activation of the Notch1 signaling promotes macrophage infiltration and strongly drives the differentiation of CD4⁺ T cells towards a Th2 phenotype, characterized by upregulated expression of IL-4 and IL-10. The Notch *γ*-secretase inhibitor dibenzazepine, by inhibiting the generation of the Notch intracellular domain and downregulating the expression of its target gene Hes1, effectively reverses the Th2 response, ultimately resulting in a significant reduction in AAA incidence and severity (69). In another study, exogenous leptin treatment markedly attenuates Ang II-induced AAA development in *ApoE^−/−^* mice by upregulating the Th1 cytokine IFN-*γ* and the transcription factor T-bet, suppressing GATA-3-mediated Th2 polarization, lowering aortic wall MMP levels, and increasing TGF-*β*1 expression, thereby stabilizing the ECM and limiting pathological remodeling (70). Although these studies do not directly confirm MMP-12 as a downstream effector, in fact, the reported protease changes primarily involve broader MMP activity or MMP-2/MMP-9. They support the concept that upstream immune polarization pathways can reshape the local protease profile during aneurysm development. Given the links between Th2 cytokines and MMP-12, future work should delineate how distinct Th cell subsets modulate macrophage polarization and MMP-12 expression, and whether these effects map onto discrete macrophage subsets rather than a simple M1/M2 dichotomy.

Nonetheless, this paradigm is not without controversy, as discordant findings suggest a more complex reality. Th1/IFN-*γ*-dependent pathways have also been implicated in aneurysm formation in both human lesions and experimental models. In human end-stage AAA lesions, infiltrating CD4⁺ T cells have been reported to display a predominant type 1 phenotype, characterized by high IFN-*γ* production and limited IL-4 expression (71). Consistently, in the CaCl₂-induced murine AAA model, CD4 deficiency or IFN-*γ* deficiency reduced aneurysm formation and decreased aortic MMP expression, whereas exogenous IFN-*γ* partially restored aneurysm susceptibility (72). These findings do not simply negate the Th2-driven model, but rather suggest that different immune programs may contribute to aneurysmal remodeling under different experimental, anatomical, or disease-stage conditions.

A more compatible explanation is that AAA-associated inflammation is not fixed in a single Th1- or Th2-dominant state, but varies across disease stage and lesion region, thereby creating distinct local cytokine milieus. In Ang II-induced AAA in ApoE⁻/⁻ mice, IL-5 and IL-10 increase early after Ang II infusion, whereas later disease is accompanied by increasing TNF-α and decreasing IL-10 (73, 74). Spatially, in late-stage AAAs, TNF-α is relatively enriched within the aneurysmal sac, whereas IL-10 remains predominant in the aneurysmal neck (74). This spatial pattern aligns with previous observations that MMP-12 specifically localizes to residual elastin fragments within the media of AAA, particularly in areas adjacent to non-dilated segments of normal aorta (50). Although these data rely on selected cytokine markers rather than complete Th-cell phenotyping, they support the broader concept that aneurysmal inflammation is temporally dynamic and regionally heterogeneous. This framework offers a cautious way to revisit why only a subset of atherosclerotic arteries undergo aneurysmal degeneration. MMP-12 expression in atherosclerotic plaques may reflect inflammatory matrix remodeling, but aneurysmal remodeling may require its convergence with a permissive cytokine milieu, an appropriate disease stage, and elastin-rich medial substrates. From this perspective, MMP-12 may represent one component of an aneurysm-prone microenvironment rather than a single determinant of the AS-to-AAA transition.

### 4.2 TGF-*β*

TGF-*β* is another central regulator of vascular homeostasis, with its signaling essential for the maintenance of normal aortic structure and function. In the canonical TGF-*β* pathway, ligands bind to receptors at the plasma membrane, leading to phosphorylation of the corresponding receptor-regulated Smads (R-Smads), namely Smad2/3 or Smad1/5/8. Phosphorylated R-Smads associate with Co-Smad (Smad4) to form a complex that translocates to the nucleus and directly regulates transcription of target genes (75).

Research over the past several decades has shown that disruption of TGF-*β* signaling promotes aortic wall destabilization, in part by derepressing MMP-12 expression and amplifying proteolytic remodeling. Systemic neutralization of TGF-*β* activity has been shown to significantly increase the incidence of aneurysm formation and rupture in a mouse model. This is accompanied by increased monocyte/macrophage infiltration into the vessel wall and upregulation of MMP-12, with the highest MMP-12 levels observed in ruptured specimens (76). Further research has corroborated and refined this perspective. In the CaCl_2_-induced AAA model, deletion of Smad3, a key intracellular mediator of canonical TGF-*β* signaling, markedly increases MMP-12, accelerates elastin degradation, and augments T-cell and macrophage infiltration. Concurrently, Smad3 deletion also activates nuclear factor-*κ*B (NF-*κ*B) and ERK1/2 signaling and increases expression of nuclear Smad2 and Smad4, as well as TGF-*β*1. Together with the concomitant rise in *in situ* gelatinolytic activity and MMP-2 and MMP-9 expression reported in the same model, these changes define a Smad3-deficiency–driven inflammatory and proteolytic program that enhances MMP-12 expression and accelerates broader ECM breakdown (77). Similarly, SMC-specific Smad4 deletion also leads to aneurysm formation and is associated with upregulation of MMP-12. Mechanistically, Smad4 deficiency promotes aortic aneurysm development through two complementary mechanisms. First, Smad4 deficiency relieves TGF-*β*–dependent repression of *MMP12* transcription within SMCs. Second, it simultaneously enhances noncanonical JNK signaling, which elevates CCL2 and drives monocyte/macrophage recruitment, thereby potentially increasing macrophage-derived MMP-12 release (78). Building on these mechanisms, a novel AAA model has been established by combining elastase-induced vascular injury with TGF-*β* neutralization. This approach recapitulates core features of human AAA: an early, self-limited dilation that progresses to sustained aneurysmal expansion in the absence of medial dissection (79).

However, a further layer of complexity is introduced by studies linking TGF-*β* signaling to immune-context-dependent vascular outcomes. In Ang II-infused ApoE⁻/⁻ mice, deficiency of the IFN-*γ*-inducible chemokine CXCL10 reduced luminal atherosclerotic plaque formation but increased aortic dilation, aneurysm severity, and rupture-related death. Notably, aneurysmal tissues in CXCL10-deficient mice were enriched for non-Th1-associated signals, including TGF-*β*1, and neutralization of TGF-*β* attenuated aortic dilation in this setting (80). These findings suggest that the vascular effect of TGF-*β* cannot be assigned a uniformly protective or deleterious role, but depends on the surrounding immune milieu and downstream signaling context.

Taken together, these data indicate that TGF-*β*/Smad signaling regulates aneurysmal remodeling through multiple, context-dependent mechanisms. In settings of impaired canonical Smad signaling, loss of TGF-*β*-mediated transcriptional restraint may enhance MMP12 expression and amplify inflammatory proteolysis. Conversely, in selected non-Th1 or CXCL10-deficient inflammatory contexts, a TGF-*β*1-enriched remodeling program may contribute to aortic dilation, indicating that the vascular consequence of TGF-*β* signaling depends on immune context, cellular targets, and downstream pathway engagement.

### 4.3 The CXCL12 (SDF-1)/CXCR4 axis

Apart from cytokine-mediated regulation, chemokine signaling, particularly the CXCL12 (SDF-1)/CXCR4 axis, also plays a pivotal role in regulating MMP-12 expression across different vascular pathologies. In AAA models, Michineau et al. demonstrated that SDF-1*α* and CXCR4 are upregulated within the aneurysmal wall and positively correlate with aortic diameter. SDF-1 is initially released by apoptotic VSMCs and subsequently by infiltrating macrophages. Crucially, pharmacological blockade of CXCR4 with AMD3100 not only inhibited macrophage recruitment but also significantly downregulated *Mmp12* mRNA levels in the aortic wall, thereby preventing elastin degradation and aneurysm expansion. This establishes a direct link between CXCR4 signaling and the transcriptional control of MMP-12 in AAA (81).

This regulatory connection appears to be conserved in AS. Marcos-Jubilar et al. observed a parallel upregulation of CXCR4 and MMP-12 protein expression in the aortas of AS mice. Furthermore, in a clinical cohort, elevated circulating levels of both SDF-1 and MMP-12 were associated with severe atherosclerosis and increased mortality risk (82). These findings imply that the SDF-1/CXCR4 axis serves as a common upstream checkpoint for MMP-12-mediated vascular remodeling in both aneurysmal and atherosclerotic diseases, likely by sustaining a pro-inflammatory macrophage phenotype characterized by high MMP-12 production. This connection not only deepens our understanding of MMP-12 regulation but also offers novel targets for the development of therapeutic strategies aimed at modulating inflammation-mediated matrix remodeling in vascular disease.

### 4.4 AP-1 as a central integrator

*MMP12* transcription is critically regulated by activator protein-1 (AP-1) signaling, with a key AP-1 binding site identified at positions -81 to -75 bp upstream of the transcription start site within the *MMP12* promoter. AP-1 comprises transcription factors from the Jun, Fos, and activating transcription factor families that typically bind DNA target sequences as homo- or heterodimeric complexes to regulate gene expression (83). GM-CSF, reported to be present in atherosclerotic plaques, enhances AP-1 DNA-binding activity and induces *MMP12* expression in monocytes (84). Mechanistically, GM-CSF signaling activates the Ras/PI3 K/Akt pathway to activate NF-*κ*B and AP-1, thereby promoting *MMP12* transcription (85). In plaque VSMCs, platelet-derived growth factor-BB upregulates *MMP12* via AP-1, whereas PI3 K inhibitors suppress transcription by blocking the PI3 K/Akt/AP-1 axis (86). Notably, PI3K-mediated regulation of MMP-12 displays cell-type specificity: in macrophages, PI3K*δ* inactivation enhances c-Jun phosphorylation and AP-1 activity, resulting in MMP-12 overexpression that drives ECM degradation and ultimately promotes aneurysm formation (87). This differential regulation may be attributed to functional divergence among PI3 K isoforms. *In vivo*, innate immune receptor signaling also appears to provide an upstream inflammatory context for sustained MMP12 expression. In human AAA tissue, TLR3 and TLR4 are highly expressed, predominantly in macrophage- and T-lymphocyte-rich regions, whereas in AngII-infused *ApoE^−/−^* mice, TRIF deficiency reduces aneurysm formation and is accompanied by lower aortic CD11b, CD68, and TNF-α expression together with a marked reduction in MMP-12, supporting a role for TLR3/4–TRIF signaling as an upstream inflammator*y* axis sustaining *MMP12* expression. AP-1-driven transcription is further subject to negative regulation by nuclear receptor signaling. PPAR*α* agonists suppress IL-1β-induced *MMP12* in human macrophages by interfering with c-Fos/c-Jun binding to AP-1 sites, whereas the PPAR*γ* agonist pioglitazone is proposed to directly bind the *MMP12* promoter to repress its expression (88, 89). Epigenetic mechanisms further modulate AP-1 access. In VSMCs, H3K9me2 enrichment at the *MMP12* promoter restricts chromatin accessibility, while inflammatory stimuli reduce global H3K9me2 and enhance NF-*κ*B and AP-1 occupancy, thereby augmenting transcription (90).

Overall, AP-1 appears to function less as a pathway-specific regulator than as a convergent transcriptional node through which cytokine, growth-factor, innate immune, chemokine, nuclear-receptor, and epigenetic signals are integrated to tune MMP12 expression. This may explain why MMP-12 induction is highly context-sensitive rather than attributable to a single upstream stimulus.

## 5 The MMP-12/EDPs module as a hub in arterial remodeling

### 5.1 MMP-12 and EDP/ERC signaling

**Figure 2:**
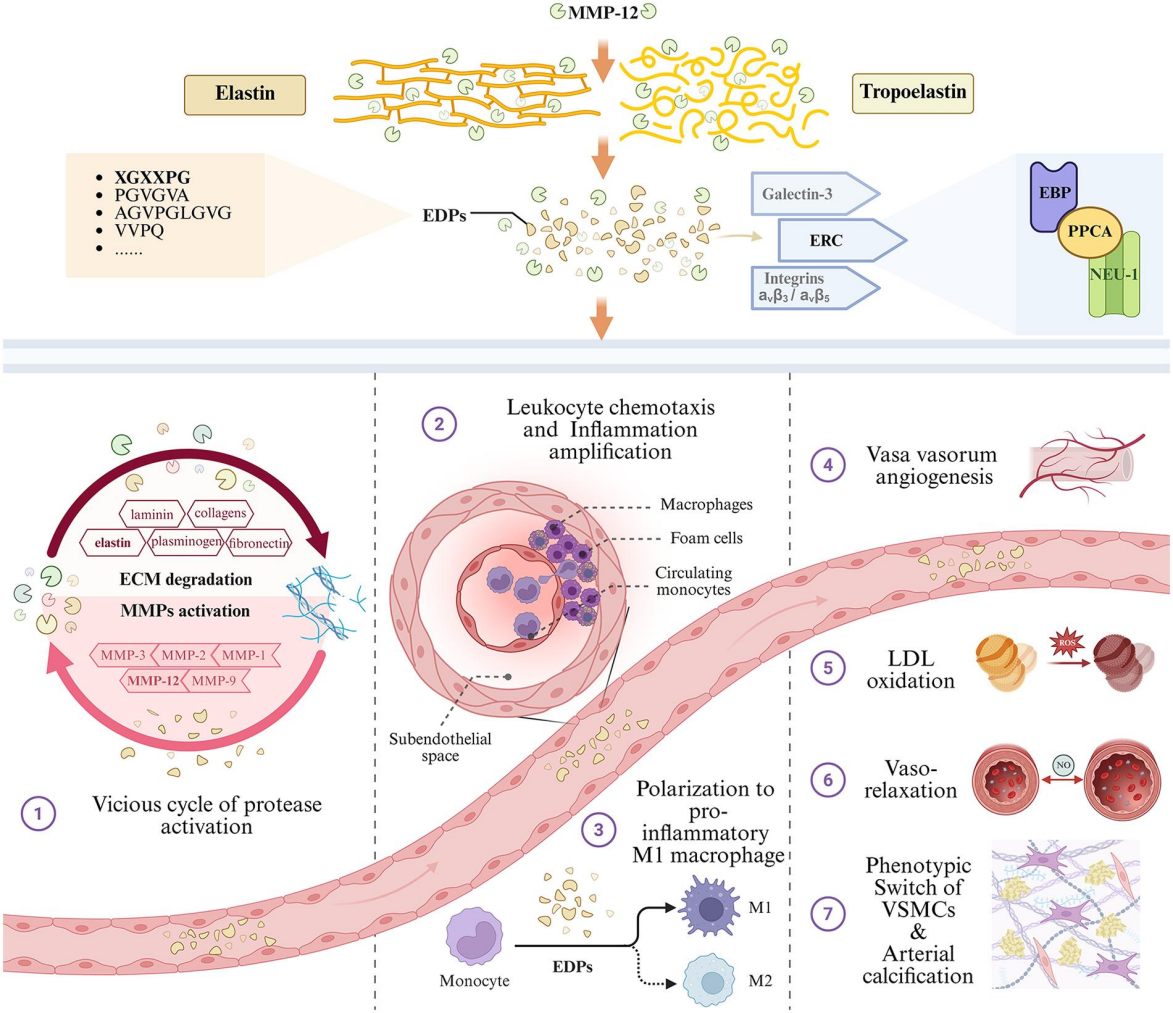
EDPs link MMP-12–driven elastolysis to self-amplifying vascular remodeling. MMP-12 degrades elastin and tropoelastin, releasing bioactive EDPs. Acting as matrikines, EDPs and sustained MMP-12 activity together establish a feed-forward pathogenic program in vascular disease. This circuitry expands proteolysis by engaging MMP activation cascades, promotes monocyte/macrophage recruitment and pro-inflammatory polarization, and is associated with vasa vasorum angiogenesis, LDL oxidation and VSMC phenotypic switching and arterial calcification, thereby accelerating maladaptive vascular remodeling. Created with BioRender.com.

As accumulating evidence indicates that MMP-12 plays a pivotal role in the initiation and progression of diverse vascular pathologies, recent studies have further revealed that its pathological effects arise not only from direct proteolysis but also from the bioactive elastin-derived peptides (EDPs) it generates. Mature elastin is a highly stable, insoluble, and hydrophobic protein formed by cross-linking of its soluble precursor, tropoelastin. Elastogenesis occurs predominantly during fetal development and infancy, providing critical elasticity and resilience to a wide range of tissues and organs. Under pathological conditions, vascular and inflammatory cells can produce tropoelastin; however, this newly synthesized tropoelastin fails to be cross-linked into mature elastic fibers (91). In chronic vascular inflammation, MMP-12 not only continuously degrades pre-existing elastic fibers but also efficiently cleaves newly synthesized tropoelastin, releasing pro-inflammatory EDPs. Relative to MMP-7 and MMP-9, MMP-12 features a larger S1’ pocket containing polar residues, conferring greater tolerance for bulky charged or aromatic amino acids at the P1’ position (92). Consistent with these features, MMP-12 can completely degrade tropoelastin within 30 min, releasing bioactive peptides enriched in VGVAPG motifs (23). In EDPs, the GxxPG motif adopts a type VIII *β*-turn conformation that is essential for downstream signaling (93). As matrikines with cytokine-like activity, EDPs exert multiple pathological effects by binding to membrane receptors. Although several receptors have been implicated, current evidence indicates that many biological effects of EDPs are mediated by the elastin receptor complex (ERC). In human cells, the ERC has been identified as a heterotrimeric complex composed of an elastin-binding protein (EBP), the membrane-associated protective protein cathepsin A (PPCA), and the membrane-bound neuraminidase Neu-1. As the core component, Neu-1 is essential for ERC signal transduction and can activate various signaling pathways that differ among cell types (94). Overall, MMP-12-mediated elastin degradation generates EDPs, which in turn signal through ERC to reinforce inflammatory activation and proteolytic responses. This creates a self-amplifying feed-forward loop that sustains multiple pathogenic processes and accelerates vascular remodeling (Figure 2).

### 5.2 MMP-12 and EDPs in macrophage recruitment and programming

Functionally, MMP-12 enables ECM proteolysis and further promotes macrophage infiltration. Shipley et al. demonstrated that *MMP12^−/−^* macrophages lose more than 95% of their elastin degradation capacity and fail to penetrate recombinant basement membranes *in vitro* and *in vivo* (95). In line with this, macrophage-specific MMP-12 overexpression in transgenic rabbits increases intimal monocyte/macrophage accumulation (38), whereas the inhibition of MMP-12 mRNA expression in macrophages by siRNA or antibody blockade reduces macrophage invasiveness by roughly 30% vs. controls (41). These data support a vicious cycle in which MMP-12-mediated degradation of elastin and basement membrane components creates space for further macrophage infiltration and foam-cell formation, thereby amplifying local inflammation and proteolysis. Furthermore, as an elastase predominantly secreted by macrophages, MMP-12 may further contribute to monocyte infiltration by generating chemotactic EDPs, although *in vivo* attribution to MMP-12 specifically remains difficult because several elastases can generate overlapping elastin fragments. Early research found that the specific hexapeptide sequence VGVAPG, present in elastin degradation, exhibits potent chemotactic activity for monocytes (96). As a truncated variant of VGVAPG, GVAPG was also found to exhibit monocyte chemotactic activity (97). Studies have demonstrated that EDPs present in AAA extracts trigger intracellular signaling cascades by binding to EBP on monocytes, thereby driving inflammatory cell infiltration. This effect was inhibited by approximately 40% following treatment with the anti-elastin monoclonal antibody BA-4 and was completely blocked by lactose (98). Subsequent research further clarified that EDPs not only act as damage signals that chemoattract monocytes/macrophages to the lesion but also modulate macrophage polarization to sustain a proinflammatory microenvironment and exacerbate vascular wall destruction (99). These findings extend the role of EDPs beyond that of mere chemotactic factors, establishing them as key modulators of macrophage inflammatory phenotypes and elucidating the underlying molecular mechanism driving the persistence of chronic inflammation in aortic disease.

### 5.3 MMP-12 and EDPs in VSMC phenotypic switching and proliferation

During early atherogenesis, medial VSMCs migrate into the intima and undergo phenotypic switching from a contractile to a synthetic state, acquiring greater proliferative and migratory capacity and enhanced matrix production that contributes to fibrous-cap formation (100). Emerging evidence positions MMP-12 as a central driver of this early remodeling. By cleaving N-cadherin, MMP-12 activates Wnt/*β*-catenin signaling to promote VSMC proliferation and intimal thickening (101). This expansion is further amplified by a Calm4-dependent AKT/MMP-12 axis (102).

In advanced plaques, however, fibrous-cap thickness and stability depend on the capacity of VSMCs to maintain collagen synthesis and a reparative phenotype. In *ApoE^−/−^/Mmp12^−/−^* mice, lesion area is reduced, with an increased proportion of VSMCs and decreased macrophage content within plaques (3). Consistently, studies of advanced human carotid plaque show an inverse association between MMP-12 and VSMC marker expression (103). Together, these findings support a stage-dependent view in which elevated MMP-12 in advanced lesions reflects a macrophage-dominant, proteolytic microenvironment that ultimately undermines plaque stability. Beyond direct matrix destruction, the proteolytic action of MMP-12 further corrupts VSMC function through the release of EDPs. By engaging the elastin receptor, these bioactive fragments drive osteogenic and calcifying reprogramming of VSMCs (104).

### 5.4 MMP-12 and EDPs in the protease cascade

Beyond its direct elastolytic activity, MMP-12 can function as an upstream amplifier of the MMP activation network. Studies demonstrate that MMP-12 can activate other MMPs such as pro-MMP-2 and pro-MMP-3 (11, 105); in turn, MMP-2 activates pro-MMP-9, while MMP-3 activates pro-MMP-1, −7, −8, −9, and −13 (106, 107). In this way, elevated MMP-12 activity broadens proteolysis from elastin to collagenous and other ECM components. Crucially, this amplification is not solely reliant on direct proteolytic activation. EDPs generated during ECM turnover also act as feed-forward signals that enhance protease expression and activation. In vascular diseases, EDPs have been shown to stimulate the release of MMPs release from human aortic endothelial cells and promote vascular matrix remodeling (108). Mechanistically, binding of VGVAPG to EBP triggers ERK signaling, thereby upregulating MMP-1 transcription (109).

Beyond these well-characterized roles, the pathophysiological contributions of EDPs to arterial diseases remain under active investigation. While EDP concentrations are typically in the ng/mL range in healthy individuals, local concentrations can surge to μg/mL at sites of active elastolysis (91). The resulting inflammatory microenvironment profoundly influences the phenotype and behavior of endothelial cells, VSMCs, and fibroblasts, driving pro-inflammatory alterations, including oxidative modification of LDL (110) and vasa vasorum formation (111).

## 6 Therapeutic strategies targeting MMP-12

### 6.1 Selective MMP-12 inhibitors

Given repeated associations between MMP-12 and pathological vascular remodeling, inhibition of MMP-12 has attracted interest as a potential therapeutic strategy in vascular disease. However, the therapeutic interpretation of MMP-12 inhibition is complicated by the conserved architecture of the MMP family. Most MMPs share a Zn^2⁺^-dependent catalytic domain, and early broad-spectrum inhibitors were commonly designed around strong zinc-binding groups, particularly hydroxamates. Although these compounds achieved potent enzymatic inhibition, they often lacked sufficient discrimination among MMP family members and therefore provided limited control over which proteolytic functions were being suppressed (112, 113).

This nonselective inhibition strategy faces significant challenges, as individual MMPs play distinct or even opposing roles in disease progression. In a murine model of brachiocephalic artery plaque instability, *Mmp12* deficiency reduced plaque area, increased VSMC content within plaques, and concurrently decreased macrophage infiltration, all of which are indicative of a more stable phenotype. In contrast, Mmp7 knockout exerted no significant effect on plaque size or stability, whereas deletion of Mmp3 or Mmp9 exacerbated plaque progression and promoted an unstable phenotype (3). Besides the risk of suppressing MMP functions involved in repair or immune resolution, lessons from earlier broad-spectrum MMP inhibitor programs have further indicated that clinical development of nonselective inhibitors was constrained by off-target inhibition of related zinc proteases, dose-limiting toxicity, and musculoskeletal adverse effects (114, 115).

The AAA field provides a disease-specific lesson of this translational difficulty. Doxycycline, a nonselective MMP-modulating tetracycline, showed mechanistic promise in experimental aneurysm models, but a multicenter randomized trial in patients with small infrarenal AAA found no significant reduction in aneurysm growth over two years (116). This neutral clinical result does not invalidate MMP biology in aneurysm disease; rather, it indicates that suppressing a broad protease signal without confirming target engagement, disease-stage suitability, or patient inflammatory phenotype is unlikely to produce reliable clinical benefit. In this setting, greater molecular precision may be needed before MMP-directed therapy can be meaningfully evaluated in vascular disease.

Structure-based inhibitor design has attempted to overcome the limitations of broad-spectrum MMP inhibition by exploiting subtle differences in MMP active-site architecture. Although the catalytic machinery is conserved among MMPs, the S1′ pocket differs in loop length, residue orientation, hydrophobicity, and conformational adaptability (117). For MMP-12, its catalytic domain features a hydrophobic, medium-sized S1′ pocket, which provides a structural basis for designing selective ligands. Among selective inhibitors studied to date, RXP470.1 has provided the most directly relevant preclinical evidence in arterial disease. Featuring a Glu-Glu motif, RXP470.1 achieves high selectivity through two complementary mechanisms. First, the Glu-Glu motif engages Thr239 and Lys177 within MMP-12's active site through specific polar interactions (118). Second, its extended hydrophobic substituent penetrates the S1′ pocket, stabilizing the binding conformation via spatial complementarity (119). This molecular design confers a 2–4 orders of magnitude enhancement in inhibitory potency against MMP-12 relative to other MMPs, reaching nanomolar affinity. In atherosclerosis models, RXP470.1 significantly reduced aortic plaque area and enhanced plaque stability, which was associated with decreased macrophage infiltration, apoptosis, and MMP-9 expression (120). In AAA models, RXP470.1 not only attenuated Ang II-induced elastic fiber fragmentation and the risk of aortic rupture but also promoted collagen regeneration to maintain vascular wall integrity, thereby restoring vascular homeostasis (5). These findings support the plausibility of selective MMP-12 blockade in arterial remodeling, while remaining limited to preclinical and model-dependent evidence.

A further limitation is that MMP-12 acts within a redundant and compensatory protease network. MMP family members share partially overlapping substrate spectra, and are co-regulated by endogenous inhibitors such as TIMPs. Moreover, zymogen activation often proceeds through sequential proteolytic cascades in which one MMP activates another (29). Therefore, blocking a single protease may merely divert proteolysis through alternative routes rather than fully suppressing matrix degradation. This network-level constraint is particularly problematic when MMP-12 is considered as a selective therapeutic target while it may not be the dominant elastase in every disease context. This point has been illustrated by earlier work. In macrophage-mediated elastolysis, MMP-12 functions as the dominant elastase when macrophages interact directly with insoluble elastin under plasminogen-free conditions. However, when plasminogen is available, plasmin-dependent activation of pro-MMP-7 can redirect elastin degradation toward a matrilysin-dependent pathway, thereby reducing the apparent dependence on MMP-12 (24). Thus, the contribution of MMP-12 is not defined solely by its intrinsic elastolytic potency, but also by the local activation state of parallel proteases. The same theme emerges from studies of IL-13-driven inflammatory remodeling, where MMP-9 and MMP-12 exhibit partially overlapping but clearly enzyme-specific effects (121). Taken together, these observations suggest that while selective MMP-12 inhibition may improve target specificity and safety, its efficacy will be highly contingent on whether MMP-12 is the dominant active protease within a given lesion, disease stage, and inflammatory-proteolytic milieu.

Beyond RXP470.1, medicinal chemistry studies have generated several MMP-12 inhibitor classes, including phosphinic peptides, arylsulfonamide carboxylates, dibenzofuran-based compounds, thioaryl derivatives, hydroxypyrone-based inhibitors, and sugar-modified water-soluble analogues (117). Clinical experience from other inflammatory diseases further supports the translational feasibility of these selective or semi-selective MMP-12 inhibitor classes for vascular disease, particularly by showing that systemic administration, short-term safety, and pharmacological modulation of MMP-12-related inflammatory pathways can be achieved in humans. AS111793, an experimental MMP-12 inhibitor evaluated mainly in respiratory inflammation models, reduced cigarette-smoke-induced airway inflammation in mice, whereas its effect was not reproduced in LPS-induced neutrophilic inflammation, indicating that MMP-12 dependence varies according to the upstream inflammatory stimulus. Similarly, dibenzofuran sulfonamide inhibitors such as MMP408, MMP145, and MMP118 have shown high potency and oral efficacy in recombinant MMP-12-driven ear-swelling, lung-inflammation, or allergen-challenge models (122–124). AZD1236, an oral dual MMP-9 and MMP-12 inhibitor, reached clinical testing in patients with COPD and showed an overall acceptable short-term safety profile (125). More recently, FP-025, also known as aderamastat, advanced the clinical feasibility of selective oral MMP-12 inhibition. In first-in-human randomized studies involving single and multiple ascending doses, FP-025 was well tolerated in healthy subjects, with repeated dosing reaching steady state by day 6 and only modest accumulation (126).

At present, selective MMP-12 inhibitors may be best regarded as mechanistic and pharmacological tools with potential therapeutic relevance in arterial disease. Available evidence indicates that these agents can help clarify the contribution of MMP-12-dependent proteolysis to vascular remodeling, while their clinical value will likely depend on the exact role of MMP-12 in vascular homeostasis. Future clinical development would require biomarker-guided patient selection rather than unstratified inhibition. Long-term safety assessment should also monitor compensatory protease activation and potential interference with MMP-12-dependent inflammatory resolution.

### 6.2 Modulation of MMP-12 by statins

The cardiovascular benefits of statins stem mainly from lipid lowering, with additional anti-inflammatory effects. MMP-12 downregulation may represent one downstream component of these pleiotropic effects. Clinically, plasma MMP-12 concentrations are higher in patients with coronary artery disease (CAD) who are not receiving statins than in healthy controls, whereas statin therapy effectively reduces circulating MMP-12 levels in CAD patients, suggesting that inhibition of MMP-12 expression may be another mechanism by which statins exert cardioprotective effects (127).

Statins regulate MMP-12 activity via both cholesterol-dependent and cholesterol-independent pathways. In the cholesterol-dependent pathway, lowering LDL cholesterol indirectly constrains MMP-12 activity. Recent studies have shown that plasma vitronectin (VTN) levels are reduced in patients with acute coronary syndrome and are negatively correlated with low-density lipoprotein cholesterol (LDL-C). Mechanistically, a high-cholesterol diet suppresses VTN promoter activity, leading to increased expression of MMP-12 in the aorta and promoting ICAM-1/VCAM-1-mediated leukocyte infiltration via activation of the NF-*κ*B pathway. By lowering circulating cholesterol, atorvastatin restores VTN expression and thereby attenuates MMP-12-driven matrix degradation within plaques (128). Independent of lipid lowering, statins also modulate MMP-12 expression through cholesterol-independent pathways. In a mouse model of atherosclerotic plaque rupture, atorvastatin reduced aortic MMP-12 activity without altering plasma cholesterol, increased plaque collagen content and fibrous cap thickness, and decreased coronary stenosis and the risk of myocardial infarction. Mechanistically, these effects were accompanied by reductions in circulating monocytes and neutrophils as well as suppression of TNF-α and IL-1β expression (129). A similar cholesterol-independent pattern has also been observed in AAA models. In an elastase-infused model, atorvastatin treatment significantly lowered aortic ICAM-1 and MCP-1 levels, limiting macrophage recruitment, decreasing MMP-12 synthesis, and reducing elastic fiber degradation, which in turn blunted aneurysm expansion (130). Importantly, elastase-induced AAA progression and its characteristic tissue pathology were not exacerbated even within a hypercholesterolemic setting, which supports the concept that the anti-inflammatory effects of statins are independent of cholesterol (131). Collectively, these findings suggest that MMP-12 modulation may represent one downstream component of the pleiotropic vascular effects of statins, supporting future exploration of novel therapeutic strategies targeting MMP-12 or its associated signaling pathways.

## 7 Conclusion

This review synthesizes the multifaceted pathological roles and mechanisms of MMP-12 in arterial diseases. By activating other MMPs and remodeling the inflammatory microenvironment, MMP-12 serves as a nodal, hub-like regulator that extends far beyond matrix degradation, driving sustained dysregulation of vascular homeostasis across diverse vascular disease phenotypes. The available evidence does not support a uniform pathogenic model. Apparent discrepancies across studies are likely driven by model- and stage-dependent differences in the dominant inflammatory milieu and substrate availability, which collectively determine the net biological output of MMP-12. At present, selective MMP-12 inhibitors are best viewed as useful mechanistic tools and potential therapeutic candidates. Their clinical utility will depend on clearer definition of MMP-12's pathological vs. homeostatic functions, the optimal timing, and the patient or lesion contexts in which target engagement is likely to be beneficial.

Several limitations should be acknowledged to guide future translation. First, much of the mechanistic evidence summarized here is derived from preclinical animal models that capture selected inflammatory programs and may not fully recapitulate the chronic, comorbidity-shaped microenvironments of human arterial disease. Second, many studies infer MMP-12 biology from transcript or protein abundance, whereas the biologically relevant variable is often compartmentalized enzymatic activity, which is shaped by local activation states, TIMP availability, and substrate accessibility. Third, causal attribution remains challenging because MMP-12 operates within a highly redundant and compensatory protease network. Overlapping substrates, compensatory upregulation of related MMPs or other proteases, and differences between genetic deletion and pharmacological inhibition can all confound interpretation of phenotype–protease relationships.

Future work should further dissect the stage-specific and cell subset–specific functions of MMP-12 and clarify how it dynamically remodels the vascular microenvironment, with the goal of enabling precise modulation of MMP-12 rather than indiscriminate inhibition. With these constraints addressed, MMP-12-based biomarkers or interventions may contribute to more precise risk stratification and mechanistically informed treatment development in selected arterial disease contexts.
